# The Impact of Authentic Leadership on Innovative Work Behavior: Mediating Roles of Proactive Personality and Employee Engagement

**DOI:** 10.3389/fpsyg.2022.879176

**Published:** 2022-06-09

**Authors:** Yina Bai, Zheng Wang, Mehboob Alam, Fozia Gul, Yiqun Wang

**Affiliations:** ^1^School of Business Administration, Liaoning Technical University, Huludao, China; ^2^School of Marxism, China University of Political Science and Law, Beijing, China; ^3^Treasurer Office, Lahore College for Women University, Lahore, Pakistan; ^4^The Institute of Management Sciences, PAK AIMS, Lahore, Pakistan; ^5^School of Continuing Education, China University of Political Science and Law, Beijing, China

**Keywords:** proactive personality, authentic leadership, work engagement, innovative work behavior, manufacturing industries 4.0

## Abstract

The purpose of the study is to investigate the impact of authentic leadership and proactive personality on innovative work behavior through dual mediation approach. This study applied a judgment sampling technique and data were gathered from 311 high-tech manufacturing industries of Shenzhen, China. The measurement model and structural model were tested using structural equation modeling technique through AMOS 24 software. The results indicate that authentic leadership has a positive and significant effect on proactive personality. Meanwhile, findings show that proactive personality has a significant impact on innovative work behavior. Moreover, findings show that proactive personality positively mediates the relationship between authentic leadership and innovative work behavior. Furthermore, findings illustrate that work engagement positively mediates the relationship between proactive personality and innovative work behavior. This study provides insightful and valuable implications to high-tech manufacturing industries executives and regulators interested in organizational productivity.

## Introduction

Existing research stated that organizations need leaders who are accomplished at motivating employees so that the employees can demonstrate positive behaviors to maintain stability and benefit the organization, especially when those behaviors are not officially stated as part of the job description (Yamak and Eyupoglu, 2021). In a competitive global market, organizations certainly face difficulties as a challenge (Ahmad et al., 2021). Authentic leadership is considered one of the most influential determinants regarding proactive personality and innovative work behavior (Hu Y. et al., 2018). Authentic leadership defines as a “leadership style that focuses on transparent and ethical leader behavior and inspires open sharing information needed to make effective and efficient decisions while accepting follower’s efforts” (Niu et al., 2018). Prior study argued that innovative work behavior defined as the employee’s attempt to offer new ideas, innovation and also support novelty to achieve organizational goals (Mubarak et al., 2021). Existing studies examined the factors that influence employee innovative work behavior (Li et al., 2016), personality traits (Hu Y. et al., 2018), servant leadership (Khan et al., 2021), and leader-member exchange (Zuberi and Khattak, 2021) in the context of behavioral context.

From the organizational perspective, a proactive personality is helpful and important to improve creativeness and generate novelty to offer useful beneficial ideas (Song and Lee, 2020). Prior study Cai et al. (2018) specifies that every individual has a diverse personality so different from each other based on personality traits. A proactive personality has a great influence on innovative work behavior and organizational performance (Newman et al., 2015). To foresee and manage a complex environment, organizations strive to establish and expand proactive behavior that ultimately improves employee affordability. Proactive personality is defined as a “comparatively established propensity to shape changes that occur in environment variation” (Seibert et al., 1999). Employees with having a proactive personality are capable to handle the situation according to requirements. They predict the scenario and react accordingly because they think in advance and explore the work setting or environment (Major et al., 2006; Fuller and Marler, 2009). Proactive personality looks at the situational scenario as an opportunity that leads them to perform in a complex situation.

Furthermore, researchers indicated that the drivers of the invention are proactive mind and personality (Seibert et al., 2001; McCormick et al., 2019). An employee’s having proactive personality keenly engages for novelty and practices that make able to think innovative ideas for the improvement and better results (Bergeron et al., 2013). Past research confirmed that improved performance is subject to the facilitation of work engagement (Bakker et al., 2008). Work engagement is associated with the positive behavior of mind at work that leads to positive work-related results (Bakker and Albrecht, 2018). An organization could establish a working setting that helps employees through the provision of job resources, societal support, and training. Employees are affected by management if they provide adequate resources as it helps them to get new services and properties. Work engagement is considered noteworthy for organizational success as they tend to be a more committed imperative resource (Bakker et al., 2008).

By comparison with other leadership styles to curtail activist psychology prior authors paid attention to the concept of authentic leadership, most in the previous decade authentic leadership has a constructive effect on employees’ behavior and attitude (Hu Y. et al., 2018; Niu et al., 2018). Authentic leadership demonstrated a transparent work environment by supporting ethical behavior, with an open mind toward change, appreciating employee interest and engagement in decisions, and promoting positive behaviors that lead to better performance (Alvesson and Einola, 2019). Authentic leadership focuses on valuable accomplishment and endorses trust in the workplace and neglects errors which resultantly provides growth of more creative ideas (Gardner et al., 2021). Authentic leaders have tolerance, open to change where employees exhibit innovative work behaviors (Avolio and Gardner, 2005).

To date, extensive research has predominantly focused on one side of the proposed relationship, that is, on how proactive personality impacts leadership and not on how leadership impacts on employee engagement with proactive personality (Bakker et al., 2012; Li et al., 2016). Authentic leadership promotes proactive behavior and engagement in leaders that reflect their positive psychological capacities on their subordinates which in turn increase employees’ psychological capital and positive emotions). Thus, authentic leadership, as a positive form of leader behavior, fosters positive emotions which can lead to change in habits and traits, resulting in a change in personality. This study is novel in that it focuses on the influence of authentic leadership on proactive personality and work engagement on innovative work behavior. This study is novel in that it focuses on the influence of authentic leadership and proactive personality, employee management on innovative work behavior.

The previous scholars argued that authentic leadership effects the proactive personality and work engagement (Wang et al., 2017; Sengupta et al., 2020). As a result, a person with proactive personality who is comparatively unrestrained by situational demands, impacting environmental change, is the best match in the constantly changing organizational setting of today. Literature review shows that proactive personality has been recognized as the significant individual-level factor in terms of influencing workers’ innovative behavior (Yamak and Eyupoglu, 2021). Therefore, this study examines the mediating effect of proactive personality and work engagement in the relationship between authentic leadership and innovative work behavior; it contributes information that will fill the gap in the literature.

The relationship between authentic leadership and innovative work behavior with the mediating roles of proactive personality and work engagement is empirically less examined in the previous literature. Yamak and Eyupoglu (2021) found a significant effect of authentic leadership on innovative work behavior in the context of the banking sector. Similarly, Mubarak et al. (2021) argued that there is a positive correlation between proactive personality and innovative work behavior. Moreover, Hu Y. et al. (2018) suggest that future researchers could take proactive personality and work engagement as a mediator to explore the innovative work behavior in the context other sectors. Therefore, this study contributes to the literature of authentic leadership, proactive personality, work engagement and innovative work behavior to answer these research questions;

RQ1. What is the role of authentic leadership style on proactive personality and innovative work behavior?

RQ2. What is the mediating role of proactive personality in the relationship between authentic leadership and innovative work behavior?

RQ3. What is the mediating role of work engagement in the relationship between proactive personality and innovative work behavior?

The next part of this paper contains a critical literature review and hypotheses development, research methodology, results, discussion, implications and limitations as well as conclusion of this research article.

## Literature Review and Hypotheses Development

### Authentic Leadership and Proactive Personality

A prior study found that authentic leadership has a positive influence on proactive personality (Guenter et al., 2017). To motivate proactive behavior in employees, leadership plays an influential role (Černe et al., 2013). Authentic leadership style provides a constructive and supportive working atmosphere that could manipulate the proactive behavior of individuals (Song and Lee, 2020). Psychological features advocate stable change in personality due to behavior sets to see a situation in a more hopeful way. Authentic leadership being an affirmative type of behavior come from leaders; produce activist thoughts in employees that help to alter their habit and behavior, resultantly personality changes. To positively affect a subordinate’s emotion and psychological ability is the illustration of authentic leadership (Alessa et al., 2021). Authentic leadership and proactive personality are positively associated with each other because authentic leaders are apt to transform and proactive personality is a trait that belongs to change the environment through leadership styles that may encourage leader’s follower’s relation (Hu et al., 2021).

In addition, authentic leadership manipulates individual various personality elements and traits that include but are not limited to psychology state, awareness of self, regulation, and moral building (Kim, 2019). Previous research by Velez and Neves (2018) argues that individuals working under the command of authentic leadership along with proactive personality positively shares new ideas. Similarly, in a work setting, the authentic leader is a role model for their subordinate declared, leader of legitimate wish to support employees self-determining philosophy for their decisions (Sengupta et al., 2020). Authentic leadership is liable to make an environment of faith and cultivate support in employees to improve self-efficacy and assure for self-competence needs. In brief, authentic leaders support employee by their words and actions and build a work environment that satisfies employee psychology and motivate them intrinsically (Wijaya, 2018). Authentic leaders are helpful toward employees to improve their proactive behavior and work-related position (Elsaied, 2018). Additionally, a positive work setup promotes employee personality traits and impacts their emotions. Thus, based on the above discussion this study posits that authentic leadership could alter the proactive behavior of employees in the workplace (Afsar et al., 2020b). So, the following hypothesis is predicted;

H1: Authentic leadership is positively related to proactive personality.

### Authentic Leadership and Innovative Work Behavior

According to prior studies, it was deduced that authentic leadership has a positive impact on the innovative work behavior of employees (Černe et al., 2013; Niu et al., 2018); because genuine leaders provide psychological assistance and psychological safety through being open, sharing, and supportive of their employees which are deemed to be critical variables in employee voice behavior. In specific, authentic leadership objectively clears data, organizes high standards of moral behavior, and fosters transparency in engaging with subordinates (Sengupta et al., 2020). By having these characteristics, leaders can reinforce their subordinates’ trust, leading to the promotion of psychological assistance and safety, and hence followers will feel free to take risks (Schuckert et al., 2018). This commitment promotes employees to convey unconventional thoughts freely and to express any view without fear. Yamak and Eyupoglu (2021) underlined that employee voice behavior, which was previously assumed to be a significantly arbitrary behavior, is proof of positive challenge that contributes to the voluntary expression of problems and propositions for innovative behaviors. Therefore, the following hypothesis is predicted;

H2: Authentic leadership is positively related to innovative work behavior.

### Proactive Personality and Innovative Work Behavior

Proactive personality is measured as a stimulus to adjust as per change and one’s capability to take action in advance according to environmental needs (Kumar and Shukla, 2019). Being proactive is associated with the constructive outcome of work, the reason being proactive personality exhibit proactive behavior (Li et al., 2020). Employees who are not proactive unsuccessful to see threats and opportunities. On the other hand, individuals with a proactive approach always search for self-improvement and other views (Neneh, 2019). A proactive personality plays a central role to motivate employees at their workplace. Individuals with proactive personalities acquire challenging tasks that give them the opportunity for novel ideas at work and other such behavior (McCormick et al., 2019). To cope with different complex situations at workplaces, proactive behavior supports a better decision, solving problems, handling a situation in advance, and offering new ideas for problem-solving. Work stress gives them sources and opportunities to develop and exercise to reduce tension (Hu R. et al., 2018).

Past researches argued that employees with proactive personalities manage work stresses and thrive in the workplace (Steinmann et al., 2018). Individuals having an active approach engaged in risk-taking and inventive so yielding innovative work behavior. Organizational competitive performance and success is aligned to proactive personality and innovative work behavior (Yamak and Eyupoglu, 2021). Build innovative work environment, proactive personality is a noteworthy attribute, on the basis of proactiveness is capable of being able to handle change business atmosphere after analyzing a complex situation or change in advance (Mubarak et al., 2021). Thus, this study proposed the following hypothesis;

H3: Proactive personality is positively related to innovative work behavior.

### Mediating Role of Proactive Personality

The past research verified the association between proactive personality and innovative work behavior (Li et al., 2016; Mubarak et al., 2021). Proactive personality as a mediator between authentic leadership and innovative work behavior is empirically less examined in the prior literature. Existing studies examined the mediating roles such as creativity (Černe et al., 2013), psychological capital (Schuckert et al., 2018) to investigate the relationship between authentic leadership and innovative work behavior. Proactive personality is a suitable mediator as it indicates a significant trait of personality that improves employee behavior (Thomas et al., 2010). Moreover, due to the volatile and changing requirement of every business especially, the manufacturing sector needs and strive to take proactive and risk-taking behavior to stay viable.

The relationship between authentic leadership and proactive personality contribute to numerous behavior and qualities that required meeting the changing demands (Wijaya, 2018). Authentic leadership promotes such personality traits in a leader’s followers’ relation. A proactive individual in a work setting holds active participation. According to Bateman and Crant (1993) proactive personality provides support, confidence, trust, hope, and spirit yielding innovative work behavior both in organizational leaders and employees. Moreover, Seibert et al. (1999) argued individuals with proactive behavior are able to handle the problem more effectively and also prepare themselves for unknown challenges (Elsaied, 2018). Individuals who hold high proactive personalities might be more innovative in the workplace (Kim, 2019). On the basis of the above discussion this study implicit that proactive personality mediates in the relationship between authentic leadership and innovative work behavior. Thus, this study hypothesized;

H4: Proactive personality mediates in the relationship between authentic leadership and innovative work behavior.

### Proactive Personality and Work Engagement

Chong et al. (2021) demonstrated that individuals having proactive personalities able to replenish personal energies to handle the situation in advance. Work engagement is defined as helpful, rewarding, job-related mind status that illustrates by vigor (energy), devotion, and absorption (interest) (Wang et al., 2017). Active and engaged workforce gives their power in form of energy to a job for getting enjoyment and activation. Being activated is a basic component of energized in a way that is alike to work engagement. Individuals having proactive behavior could be more engaged in work, due to refilling energy that helps to motivated and animated at work (Lv et al., 2018). Additionally, engagement is defined by Bakker et al. (2012) as an emotional drive that represents an employee’s mindset about the job. Work engagement is associated with the individual job satisfaction to stay connected with the organization for a longer period of time (Yang et al., 2017). Accordingly, this study predicted the following hypothesis;

H5: Proactive personality is positively associated with work engagement.

### Work Engagement and Innovative Work Behavior

There is a positive relationship between innovation and engagement, particularly engaged individuals to exhibit innovative work behavior (Agarwal, 2014a). It is suggested by Koroglu and Ozmen (2022) for extra-role performance, the first moment is for employees to be engaged in their current role and organization. To perform in the extra role, they act in experimental view which leads them for novel idea generation and solution to complex problems in an innovative manner, called innovative work behavior (Afsar et al., 2020a). Innovative and creative individuals are able to offer new ideas for self-motivation by realizing the work environment. Support from the employer is required for the realization of ideas. The prior study argued that to generate new ideas for innovative work and engaged employees provide better support and worth to their work (Agarwal, 2014b). Innovative work behavior is inclined with employee engagement as they are well aware regarding the workplace psychologically, physically, and cognitively (Černe et al., 2013).

Innovative work behavior introduces, application and understanding of novel ideas, products, procedures, processes to the individual role, group, and organization (Li et al., 2016). The association between work engagement and innovative work behavior is acknowledged in previous research that explained individuals with a high level of engagement are more likely to perform efficient work, involve in innovative ideas, and exhibit new kinds of solutions to overcome problems (Afsar et al., 2020a; Slåtten and Mehmetoglu, 2011) found that to get new ideas at the workplace, innovative work behavior is an appropriate function. Engaged employees tend to be more active with a greater level of power and more passionate regarding jobs. Work engagement is greatly linked to innovative work behavior. A diverse sample of work engagement among members along with innovative ideas recognized by Seppälä et al. (2018). Thus, employee innovation and innovative work behavior boost a high degree of engagement and reduce short engagement altitude. Hence, the proposed hypothesis is predicted;

H6: Work engagement positively related to innovative work behavior.

### Mediating Effect Work Engagement

For any organization, employees are valuable resources and concerned about their well-being for establishing a healthy and conducive work atmosphere (Yamak and Eyupoglu, 2021). Numerous studies confirmed that work engagement contains a degree of motivation and enthusiasm, with commitments toward job tasks (Buil et al., 2019). According to Lai et al. (2020) work engagement hold engagement in various shapes (e.g., trait engagement, behavior engagement, and state engagement), to confirm that engagement characteristic is unlocked for different theoretical and practical research.

To achieve extra performance in an organization, proactive individuals face challenges in representing their novel initiatives and struggle to see barriers and problems as an opportunity (Young et al., 2018). Improvement in engagement gives proactive behavior at work in form of personal initiatives. Buil et al. (2019) remarked that individuals with proactive personalities are vigorously engaged in assigned duty and citizenship behavior. Prior studies argued that proactive personalities tend to be more passionate regarding work, complex job duties, and handling of resources in a more effective manner with new ideas to establish work engagement (Koroglu and Ozmen, 2021; Mubarak et al., 2021). Moreover, existing studies discovered that performance at a job and personal behavior (i.e., proactive personality, self-efficacy) are best predictors regarding employees’ work engagement because it wraps every aspect of employee effort for goal achievement (Cai et al., 2018; Sengupta et al., 2020).

Behavioral engagement is a character of work engagement, considered the most significant trait obligatory to be successful in business, magnifies innovative work behavior amongst employees (Wang et al., 2017). Therefore, organizations take special concern regarding the work engagement of employees to get an acceptable stage to accomplish innovative work behavior. According to Koroglu and Ozmen (2022) for an organization’s sustainability in the long term, innovative work behavior is crucial, hence reflecting the need to get employee innovative work behavior via proactive personality and work engagement. In a global market, an organization can add value and competitiveness by establishing achieving innovative work behavior. Innovation could give chance to every employee to offer their creative thoughts for the enhancement in work setting engagement (Agarwal, 2014b). Thus, based on the discussion, this study predicted the following hypothesis;

H7: Work engagement mediates between proactive personality and innovative work behavior.

### Conceptual Model

**FIGURE 1:**
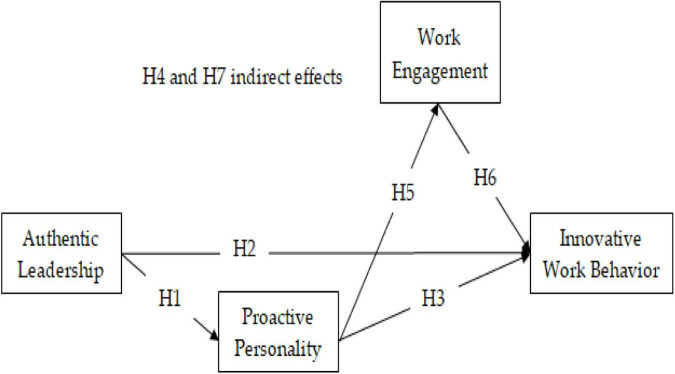
Conceptual Model.

The conceptual model depicting the relationships and hypothesis is given in Figure 1.

## Methodology

### Sample Technique and Procedures

The nature of this study was cross-sectional and the target population was included the high-tech manufacturing industries of Shenzhen province of China. This study applied non-probability judgment sampling technique to determine the samples. This study used primary data with the help of open and close ended questionnaire. The data was collected from top, middle and lower level employees of high-tech manufacturing industries of China. Before going to collect data from respondents some procedures were adopted. Firstly, we sent the permission letter to all the manufacturing industries that are currently involved in making high-tech machinery and products. Secondly, we clearly discussed the aim and importance of this study among respondents to engage them in data collection process. After the approval from the industries, top, middle and lower management employees were invited to participate in this study. The original draft of the questionnaires was in English language and translation was checked by using the translation and back-translation process through the two language experts who have good command on Chinese and English language.

The employees were free to respond, and no incentive was offered. Initially, 400 paper-pencil questionnaires were distributed among employees and 331 responses were returned by the respondents and the participation rate was 82.5%. Moreover, 20 responses were incomplete and then discarded. Thus, the final response size compromised of 311 questionnaires. Among the valid responses, 275 (88%) respondents were male, and the remaining 36 (12%) were females. 127 (41%) respondents had a graduation degree which is the highest and 4 (1.3%) respondents had post-graduate education which was the lowest whereas the other 180 (57.8%) fall in between. The 95 (31%) respondents belong to the age group (20-31) which are the highest and 59 (19%) are belong to the age group (41–50) which are the lowest where the others 157 (50%) age group. Respondents 125 (40%) had experience range (0-5) which were highest and 10 (3.2%) which are lowest and others 176 (57%) belong to another experience group.

Furthermore, Harman’s single factor was applied to test common method bias CBM in the data. According to this methodology, all the factors are merged in factor analysis and the first factor explained more than 50% of the total variance that means there is an issue of CBM in the data. The findings from factor analysis indicate that, the first factor explained 32.82% (< 50%) of the total variance (Podsakoff et al., 2003). Thus, there is no issue of common method bias in the data.

### Instruments

This study adopted the measurement scales from the already published work of prior researchers on the constructs such as authentic leadership, proactive personality, work engagement and innovative work behavior. Moreover, a five-point Likert scale ranging from (strongly disagree 1, to 5 strongly agree) except control variables was used to assess the response of participants.

#### Authentic Leadership

To measure authentic leadership, we used eight items from the prior study by Walumbwa et al. (2007). This scale was used by prior researchers to identify the impact of authentic leadership on innovative work behavior (Yamak and Eyupoglu, 2021). A sample item “Seeks feedback to improve interactions with others.” The Cronbach’s alpha for authentic leadership was 0.920.

#### Proactive Personality

To assess the proactive personality, we used five measurement items from the previous study by Janssen et al. (2017). A sample item “I excel at identifying opportunities.” The Cronbach’s alpha for proactive personality was 0.920.

#### Work Engagement

To evaluate the work engagement, we used nine items from the study by Schaufeli et al. (2006). This scale was adopted by prior researchers to examine the work engagement of employees (Sengupta et al., 2020). A sample item is “I get carried away when I am working.” The Cronbach’s alpha for work engagement was 0.937.

#### Innovative Work Behavior

To assess the innovative work behavior, we used six measurement constructs from the study by Scott and Bruce (2017). A sample item “Generates creative ideas.” The Cronbach’s alpha for innovative work behavior was 0.919.

### Statistical Methods

This study analyzed confirmatory factor analysis, reliability and validity analysis and structural model analysis with the help of statistical SPSS and AMOS software’s. As suggested by prior researcher SPSS and AMOS software’s were provided suitable results to test the direct and indirect hypotheses relationships (Hayes and Preacher, 2013).

## Results

### Measurement Model

The confirmatory factor analysis (CFA) was performed through AMOS software and findings were shown in Table 1.

**TABLE 1:** Measurement model analysis.

Constructs	Items	Measures	Std. β	S.E	Z	*p*
Authentic leadership						
	AL 1	Seeks feedback to improve interactions with others.	0.926	−	−	−
	AL 2	Accurately describes how others view his or her capabilities.	0.845	0.035	25.613	***
	AL 3	Says exactly what he or she means.	0.860	0.034	26.814	***
	AL 4	Is willing to admit mistakes when they are made.	0.813	0.035	23.500	***
	AL 5	Demonstrates beliefs that are consistent with actions.	0.834	0.037	24.892	***
	AL 6	Makes decisions based on his/her core beliefs.	0.849	0.034	25.949	***
	AL 7	Solicits views that challenge his or her deeply held positions.	0.737	0.045	19.325	***
	AL 8	Listens carefully to different points of view before coming to conclusions.	0.834	0.035	24.886	***
Proactive personality						
	PP 1	Wherever I have been, I have been a powerful force for constructive change	0.827	−	−	−
	PP 2	If I see something I don’t like, I fix it.	0.903	0.052	22.799	***
	PP 3	I love being a champion for my ideas, even against others opposition.	0.845	0.055	20.552	***
	PP 4	I excel at identifying opportunities.	0.822	0.056	19.671	***
	PP 5	I can spot a good opportunity long before others can.	0.891	0.053	22.335	***
Work engagement						
	WE 1	At my work, I feel bursting with energy.	0.843	−	−	−
	WE 2	At my job, I feel strong and vigorous.	0.796	0.044	19.533	***
	WE 3	I am enthusiastic about my job.	0.842	0.045	21.425	***
	WE 4	My job inspires me.	0.782	0.043	18.999	***
	WE 5	When I get up in the morning, I feel like going to work.	0.796	0.046	19.534	***
	WE 6	I feel happy when I am working intensely.	0.833	0.045	21.037	***
	WE 7	I am proud of the work that I do.	0.857	0.046	22.075	***
	WE 8	I am immersed in my work.	0.876	0.044	22.912	***
	WE 9	I get carried away when I am working.	0.792	0.044	19.365	***
Innovative work behavior						
	IWB 1	Searches out new technologies, processes, techniques, and/or product ideas.	0.862	−	−	−
	IWB 2	Generates creative ideas.	0.888	0.045	24.388	***
	IWB 3	Promotes and champions ideas to others,	0.887	0.045	24.313	***
	IWB 4	Investigates and secures funds needed to implement new ideas.	0.866	0.044	23.219	***
	IWB 5	Investigates and secures funds needed to implement new ideas.	0.821	0.046	21.108	***
	IWB 6	Is innovative.	0.831	0.044	21.555	***

***Significant (p < 0.001).

Moreover, Table 2 findings indicate that four measurement constructs have acceptable reliability results, because all the values of Cronbach’s alpha exceeded 0.70 and the composite reliability ranged from (0.933 to 0.950) surpassed the recommended benchmark of 0.80 (Bagozzi, 1977). Furthermore, regarding the validity test, the entire item loadings ranged from (0.737 to 0.926). The values of the AVE’s were also acceptable and ranged from (0.680 to 0.736). Additionally, to evaluate the discriminant validity, we used criteria given by Fornell and Larcker (1981). Table 2 results depict that the measurement model has satisfactory outcomes because square root of the AVE were greater than the values of its corresponding row and columns. For the goodness-of-fit index results showed as follows; The values for (Chi-square X^2^ = 929.912; df = 344; X^2^ = /df = 2.701; CFI, 0.942; NFI = 0.911; GFI, 0.857; RMSEA, 0.052 and RMR, 0.033) as recommended values by Hu and Bentler (1999) and Hayes (2013). Thus, all the measurement values were acceptable.

**TABLE 2:** Reliability and validity analysis.

	CA	CR	AVE	WE	AL	IWB	PP
WE	0.949	0.950	0.680	**0.825**			
AL	0.944	0.950	0.704	0.257***	**0.839**		
IWB	0.940	0.944	0.738	0.222***	0.231***	**0.859**	
PP	0.930	0.933	0.736	0.298***	0.286***	0.292***	**0.858**

*AL = Authentic leadership; PP = Proactive personality; WE = Work engagement; IWB = Innovative work behavior.*

*Values diagonals with bold are the square root of the AVE.*

*Values are under diagonals are the correlations.*

***Significant (p < 0.001).

### Structural Model and Hypothesis Testing

**FIGURE 2:**
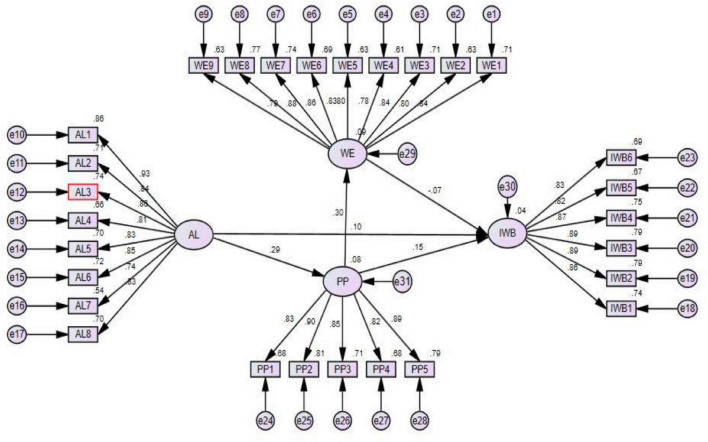
Structural Model.

To test the hypothesis we applied structural equation modeling technique for testing the direct and mediation effect suggested by Hayes (2013). The results were shown in Table 3 and Figure 2. Findings show that authentic leadership had a positive and significant effect on proactive personality (β = 0.287^***^, *Z* = 5.524, *p* < 0.002). Therefore, H1 was supported. Moreover, results indicate that authentic leadership had a significant impact on innovative work behavior (β = 0.097^***^, *Z* = 1.999, *p* < 0.047). So, H2 was supported. Meanwhile, we found that proactive personality had a significant influence on innovative work behavior (β = 0.155^***^, *Z* = 2.672, *p* < 0.008). Therefore, H3 was accepted. Furthermore, findings indicate that proactive personality had a significant impact on work engagement (β = 0.300^***^, *Z* = 5.696, *p* < 0.000). Thus, H5 was supported. Additionally, we found that work engagement had an insignificant effect on innovative work behavior (β = −0.070, *Z* = −1.276, *p* < 0.202). Therefore, H6 was not accepted.

**TABLE 3:** Direct effects.

Hypotheses	Direct Relationships	Un-standardized Estimates	S.E	Critical Ratio	*p* value	Standardized Estimates
H1	AL→PP	0.301	0.055	5.524	0.002	0.287***
H2	AL→IWB	0.090	0.050	1.999	0.047	0.097***
H3	PP→IWB	0.136	0.051	2.672	0.008	0.155***
H5	PP→WE	0.283	0.050	5.696	0.000	0.300***
H6	WE→IWB	−0.065	0.051	−1.276	0.202	−0.070

*AL = Authentic leadership; PP = Proactive personality; WE = Work engagement; IWB = Innovative work behavior.*

Significant < 0.001***.

Besides, Table 4 presented the results of the mediation analysis. To assess the indirect effect of proactive personality and work engagement, the bootstrap method was applied at a 95% confidence interval with 5000 bootstrap samples. We followed the recommendations by Hayes and Preacher (2013) to evaluate the confidence interval of the lower and upper bounds of percentile method to analyze whether indirect effect was significant or not. The standardized indirect effect show that proactive personality partially mediates in the relationship between authentic leadership and innovative work behavior (β = 0.038^***^, *p* < 0.000). Therefore, H4 was significant. Lastly, the standardized indirect effect show that work engagement partially mediates in the relationship between proactive personality and innovative work behavior (β = 0.086^***^, *p* < 0.000). Hence, H7 was also supported and mediation was significantly proved.

**TABLE 4:** Indirect effects.

Path Coefficients and Hypotheses	Std. Estimations	Bootstrapping 5000 samples with a 95% Confidence Interval	*P Value*
		Percentile Lower and Upper	
**H4**			
Standardized Direct Effects			
AL→IWB	0.287***	0.090, 0.350	0.001
Standardized Indirect Effects			
AL→PP→IWB	0.038***	0.020, 0.120	0.001
Standardized Total Effects	0.325***	0.200, 0.400	0.001
**H7**			
Standardized Direct Effects			
PP→IWB	0.155***	0.120, 0.210	0.001
Standardized Indirect Effects			
PP→WE→IWB	0.086***	0.030, 0.130	0.001
Standardized Total Effects	0.241***	0.190, 0.320	0.001

*AL = Authentic leadership; PP = Proactive personality; WE = Work engagement; IWB = Innovative work behavior.*

Significant < 0.001***.

## Discussion

This study attempts to address the imperative gap that exists in previous works by examining the impact of authentic leadership, proactive personality on innovative work behavior in the Chinese manufacturing industries context. This study found authentic leadership as a situational variable works as a predictor between proactive personality and innovative work behavior. This result is matched with previous research (Ahmad et al., 2021). In past decades, the theory of authentic leadership is powerful that offers support regarding a leader’s impact on followers. Similarly, encouraging affiliation has been recognized among authentic leadership and followers’ behavior. Alike, authentic leadership facilitates the improvement of employees’ capabilities, self-esteem, and self-efficacy. Consequently, authentic leadership plays an important role among proactive personality and innovative work behavior.

This study found that proactive personality mediates in the relationship between authentic leadership and innovative work behavior. Moreover, our results show that proactive personality had a significant influence on innovative work behavior as well as work engagement positively mediates in the relationship between proactive personality and innovative work behavior. This study’s results are noteworthy as it expands previous research through an explanation of authentic leadership and proactive personality that manipulate employee innovative work behavior. Our study findings are similar to a prior study by Yamak and Eyupoglu (2021) who recommended that the role of authentic leadership provides a significant result for the provision of skilled and well-groomed employees to an organization.

This study finding indicates that proactive personality and work engagement significantly supports employee innovative work behavior. So, in the present viable business setting, this study suggests organization inducts individuals that hold proactive personalities or a good working atmosphere that develops their personalities. This finding is in line with existing research by Mubarak et al. (2021). Induction of employees with proactive personalities is smart and capable those give better results, so organization should retain them. Such employees prove to be assets for an organization. Our findings are aligned to past research that shows organizations increasingly depend on resourceful employee actions to amend bottom-up actions to better deal changing environment (Li et al., 2016).

Furthermore, this study found that work engagement positively mediates the relationship between proactive personality and innovative work behavior. This result of this relationship is similar to a prior study (Hu et al., 2021), which suggests that engaged employees offer better results and an effect on the performance of the organization as well as innovative work behavior. Employees holding well-built personal resources as proactive personalities might be notably engaged in their job. A proactive person describes personal ideas in a wide variety of actions and situations. Employees with proactive personalities tend to see opportunities, take initiative, and be risk-takers. They are capable to recognize opportunities to bring positive changes in their personality.

## Implications and Limitations

This study provides some theoretical and practical contributions. Initially, the current paper verifies the positive association among authentic leadership, proactive personality, and work engagement as well as innovative work behavior. This research proved the positive direct effect of authentic leadership on proactive personality and the mediation effect of proactive personality in the relationship between authentic leadership and innovative work behavior. Additionally, this research established the direct effect of proactive personality on work engagement and mediating effect of work engagement in the relationship between proactive personality and innovative work behavior. To meet the present challenges of a competitive business scenario, behavior like innovation at work is essential. So, organizations must center on individual personalities and preferred individuals with proactive personalities or boost their personality in the course of training. Moreover, this paper suggested that organizations must take care and concentrate on employees’ work engagement. Employee work engagement offers novelty; inventiveness transports new innovative ideas that can be used for better work output from employees for enhanced individual and organizational performance.

This study finding has important suggestions and recommendations for the high-tech manufacturing industries of China. First, authentic leadership actions that the subordinate perceives as courteous and polite should be established. Leaders should concentrate on stimulating motivation behaviors that allow followers to feel treated honestly and consistently based on ethical and moral standards in order to improve innovative work behavior. These findings could be used in leadership development programs. Understanding the psychological mechanisms that underpin transformative leadership is critical for increasing productivity in real-world contexts.

Second, given the favorable effect of proactive personality on employee work engagement and innovative work behavior, leaders could use a screening technique to examine candidates’ personality traits during selective staffing and recruit the individuals whose personality best suits the frontline service roles. Candidates with a personality attribute like proactive personality should be chosen using a combination of proactive personality surveys and modeling real-life scenarios at work and seeing how employees react. Thirdly, leaders should support proactive behavior in their staff by creating an environment that encourages it. Organizing proactive training programs where employees are aware of various ways to develop their traits in the workplace and are instructed on how to remodel their working environments and address problems at work in a proactive manner. These proactive training programs should be based on previous occurrences in order to prepare personnel for real-life scenarios that can be repeated.

Furthermore, leaders may improve their competitive edge by attracting and retaining people who take the initiative to not only do their jobs but also to help others. This study result shows that engaged of proactive personality has higher work engagement and innovative work behavior, both of which are critical in the competitive high-tech manufacturing sector. Employees can cultivate various strategies to become more engrossed in their work through engagement development programs or workshops, or by establishing a clear performance goals-system within the organization to further motivate employees and improve individual job performance.

This study has some limitations that would be considered for future research opportunities. First, the design of cross-sectional data was used, so future research could use time-lagged data. Second, a future researcher can collect data from a large population as this study used a small sample size. Third, data is collected from a specific province of Shenzhen, China that could cause generalization of results, so the future study could examine in other regions or countries. This study does not measure of leader’s demographic, which might influence attitude and style of leadership. A future researcher may look into another leadership style to better evaluate employees’ innovative work behavior at work setup.

## Conclusion

This article explores the effect of authentic leadership on proactive personality and innovative work behavior along with the indirect effects of proactive personality and work engagement. The data of this study were retrieved from the high-tech manufacturing industries of Shenzhen, China. Results conclude that authentic leadership has a positive impact on proactive personality and innovative work behavior. In addition, proactive personality and work engagement partially mediates in the relationship between authentic leadership and innovative work behavior. Most of the research related to authentic leadership has been conducted on populations and samples in Western countries a fact which initiated the implementation of the present study to develop the authentic leadership construct within different cultural context to enhance the generalizability of the resultant authentic leadership’s measure.

## Data Availability Statement

The raw data supporting the conclusions of this article will be made available by the authors, without undue reservation.

## Ethics Statement

The studies involving human participants were reviewed and approved by China University of Political Science and Law. The patients/participants provided their written informed consent to participate in this study.

## Author Contributions

YB and ZW proposed the research and wrote the manuscript. MA, FG, and YW designed and carried out the methodology, results and extensively edited the manuscript. All authors contributed to the article and approved the submitted version.

## Conflict of Interest

The authors declare that the research was conducted in the absence of any commercial or financial relationships that could be construed as a potential conflict of interest.

## Publisher’s Note

All claims expressed in this article are solely those of the authors and do not necessarily represent those of their affiliated organizations, or those of the publisher, the editors and the reviewers. Any product that may be evaluated in this article, or claim that may be made by its manufacturer, is not guaranteed or endorsed by the publisher.
